# Daily Physical Activity Does Not Contribute to Differences in Muscle Oxidative Capacity Between Overweight and Obesity

**DOI:** 10.1002/edm2.513

**Published:** 2024-08-14

**Authors:** Abigayle B. Simon, Cassandra C. Derella, Jacob C. Looney, Kimberly Norland, Xiaoling Wang, Ryan A. Harris

**Affiliations:** ^1^ Georgia Prevention Institute, Medical College of Georgia Augusta University Augusta Georgia USA

**Keywords:** adiposity, daily activity, mitochondrial function, skeletal muscle

## Abstract

**Background:**

The interaction between physical activity, skeletal muscle health, and adiposity has been explored in normal weight and overweight/obesity grouped together; however, the overall risks associated with being overweight are less than those observed with obesity and can be confounded by disparities in both sex and race. Thus, the present study sought to investigate the intricate interplay of daily physical activity and skeletal muscle oxidative capacity (SMOC) in overweight and obesity, while exploring how sex and race impact this dynamic relationship.

**Methods:**

One hundred and forty participants were grouped by body mass index (BMI) as overweight (*n* = 73; BMI >25–<30 kg/m^2^) or obese (*n* = 67; BMI ≥30 kg/m^2^). SMOC was assessed using near‐infrared spectroscopy and daily physical activity was assessed for 7 days using accelerometry.

**Results:**

Overweight individuals exhibited a higher (*p* = 0.004) SMOC and engaged in more (*p* = 0.007) vigorous physical activity compared to obese individuals. In addition, SMOC was lower (*p* = 0.005) in obese non‐Hispanic Black (NHB) men compared to overweight NHB men. No relationships between physical activity and SMOC were observed.

**Conclusion:**

Physical activity is not associated with differences in SMOC in overweight and obesity. Obese individuals engage in less vigorous physical activity and exhibit lower SMOC compared to overweight individuals and these differences are emphasised in NHB men.

## Graphical abstract

Overweight individuals exhibit higher skeletal muscle oxidative capacity and engage in more vigorous physical activity compared to their obese counterparts. No correlation between physical activity and muscle oxidative capacity were observed and suggest that factors beyond increased activity contribute to greater muscle oxidative capacity in overweight versus obese individuals.

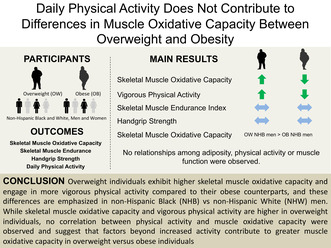

## 1 Background

The prevalence of obesity has surged almost threefold in the last five decades, and this upward trajectory is continuing to rise [1]. Currently, 36% of Americans are considered overweight with a body mass index (BMI) between 25 and 30 kg/m^2^, while 33% of individuals have a BMI over 30 kg/m^2^ and are considered obese [2]. These staggering statistics have lead some experts to characterise increased adiposity and obesity as a 21st century epidemic [3]. In fact, the economic impact of obesity places a substantial financial burden on the United States, equating to a staggering $160 billion annually [4]. While even slight increases in adiposity pose additional health risks, the risks associated with being overweight are less than those observed with obesity [5, 6]. Importantly, however, the prevalence of hypertension [6] and diabetes [7] is increased in individuals who are overweight compared to those who are normal weight. Perhaps somewhat unsurprisingly, the rates of obesity disproportionately impact both women and non‐Hispanic Black (NHB) individuals, both populations who exhibit a greater prevalence of obesity compared to men and non‐Hispanic White (NHW) individuals [8].

Physical activity encompasses body movements generated by skeletal muscles, leading to energy expenditure. Exercise, on the other hand, is a subset of physical activity, characterised by planned, structured activities aimed at improving physical fitness [9]. Physical activity has been recognised as both a treatment and prevention strategy to not only reduce body mass, but also attenuate the accumulation of adipose tissue [10]. In fact, increasing daily physical activity has been associated with a lower risk cardiovascular disease (CVD) risk, cancer mortality and all‐cause mortality [11]. While there are many physiological benefits of physical activity, disparities in physical activity patterns exist among sex and racial groups and these differences may help explain the health disparities observed in obesity. In general, men and NHW individuals engage in higher rates of physical activity compared to women and NHB individuals [12, 13]. Additionally, normal weight individuals engage in a higher frequency of both moderate and vigorous physical activity compared to obese individuals [14]. Further, individuals with a higher socioeconomic status (SES) engage in more leisurely physical activity, albeit no relationship identified between SES and occupational and transport physical activity [15]. Despite a plethora of existing research comparing normal weight to obesity, there is limited evidence, if any, that has specifically focused on the comparison of physical activity patterns between overweight and obese individuals.

Obesity is a chronic inflammatory condition that not only contributes to various pathological conditions [16], but also negatively affects skeletal muscle health [17]. Mitochondrial dysfunction has emerged as a key contributor to the pathogenesis of obesity, due to the role of skeletal muscle as an essential metabolic regulator [18]. Indeed, increased adiposity can precipitate a decline in the contractile function of skeletal muscle [19], thereby leading to skeletal muscle complications, such as glycemic instability, a phenotype that is commonly observed in obesity. In addition, the combination of increased adiposity and lower fat‐free mass contributes to insulin resistance, a state that not only negatively impacts glucose uptake, but can also result in skeletal muscle fatigue [20]. Consequently, the reduction in fat‐free mass can indirectly contribute to an increase in percent body fat [21]. Sex and race disparities in skeletal muscle physiology also exist. Specifically, men and NHB individuals exhibit greater skeletal muscle function compared to women and NHW individuals [22, 23]. Despite this, the exact mechanism underlying race and sex differences in skeletal muscle oxidative capacity (SMOC) is currently unknown. Moreover, current literature highlights differences in skeletal muscle health between normal weight and obese individuals [24]; however, whether or not SMOC is different between overweight and obese individuals has yet to be elucidated.

There is certainly a known positive relationship between physical activity and skeletal muscle health [25, 26]. Physical inactivity disrupts mitochondrial plasticity, promoting a decrease in both mitochondrial size and number, as well as functionality [27]. Physical inactivity reduces mitochondrial biogenesis and contributes to mitochondrial dysfunction [28]. Further, increased physical activity provokes a transformation in skeletal muscle fibres towards more slow‐oxidative or Type 1 muscle fibres [29], which are rich in mitochondria and contribute to enhanced oxidative function. The interaction between increased adiposity, muscle oxidative capacity and physical activity is extremely complex. Whether physical activity affects SMOC in overweight and obese individuals has yet to be investigated. Accordingly, the present study sought to investigate the intricate interplay of daily physical activity and SMOC in overweight and obesity, while exploring how sex and race (NHW and NHB) impact this dynamic relationship. It was hypothesised that individuals who were obese would demonstrate lower daily physical activity and SMOC compared to individuals who were overweight.

## 2 Methods

### 2.1 Experimental Design

All participants reported to the Georgia Prevention Institute at Augusta University following an overnight fast and having abstained from caffeine, smoking or any tobacco use or moderate‐to‐vigorous exercise for 12 h prior to testing. The visit consisted of the informed consent process, body composition measurements, anthropometric measures and SMOC testing. Height and body mass were determined using a stadiometer and standard platform scale (CN20, DETECTO, Webb City, MO), respectively, and were used to calculate BMI. Total body fat was determined using dual energy X‐ray absorptiometry (QDR‐4500W; Hologic, Waltham, MA). A single stick blood draw was performed to obtain clinical laboratory values, including fasting glucose, fasting insulin, haemoglobin A_1c_ and a complete lipid panel. Homeostatic Model Assessment for Insulin Resistance (HOMA‐IR) was calculated using the following equation: HOMA‐IR = (insulin × glucose)/405. Physical activity was monitored for 7 days following the testing date.

### 2.2 Participants

The study was conducted in Augusta, GA, and surrounding areas, which consist of 56.4% NHB individuals, with 70% of the population being either overweight or obese. Consequently, 140 participants (24 twin pairs and 92 singletons; men: *n* = 54, women: *n* = 86; NHW: *n* = 69, NHB: *n* = 71) from the community were recruited as part of an ongoing, longitudinal twin cohort parent study. The participants were classified as either overweight (*n* = 73) or obese (*n* = 67) by the National Institute of Health's definition of a BMI of >25–<30 kg/m^2^ and ≥30 kg/m^2^, respectively [30], and subsequently grouped by their self‐reported race as either NHW or NHB. Participants were excluded if they (1) were currently pregnant or nursing; (2) had a previous diagnosis of major cardiovascular disease including myocardial infarction, congestive heart failure or stroke; (3) had a previous diagnosis of major lung disease including chronic obstructive pulmonary disease; (4) had a previous diagnosis of cancer; or (5) had a haemoglobin HbA_1c_ ≥6.5%. All study protocols were approved by the Institutional Review Board at Augusta University (IRB: 1323570).

### 2.3 Skeletal Muscle Health

#### 2.3.1 Skeletal Muscle Endurance Capacity

Skeletal muscle endurance, integral to muscular fitness, encompasses the capacity for sustained activity and resistance to fatigue during prolonged contractions [31]. A maximal tolerable electrical stimulation (Winner/ST EVO, Richmar, Clayton, MO) was determined for each participant, and used to evoke 5 min of involuntary twitch contractions of the gastrocnemius at 5 Hz as previously described. A 6‐axis, wireless accelerometer (Metamotion RL, Mbientlab, San Jose, CA) was placed on the belly of the medial gastrocnemius to capture surface oscillations throughout the 5‐min maximal muscle contraction [32]. By identifying peak and end twitch acceleration points, a skeletal muscle endurance index was calculated as previously described [32, 33, 34, 35]. A higher endurance index is indicative of greater muscle endurance and lower muscle fatigability and provides a quantitative measure of sustained muscle performance.

#### 2.3.2 Skeletal Muscle Oxidative Capacity

Following the skeletal muscle endurance test, SMOC was assessed non‐invasively by measuring the rate of muscle oxygen consumption following an increase in muscle metabolism induced by electrical stimulation, as previously described by our team [36, 37] and others [38, 39]. Briefly, the participant laid in a semi‐recumbent position with their right leg positioned in an immobilised position. Near‐infrared spectroscopy (NIRS; PortaLite Artinis Medical Systems, Netherlands) was placed on the belly of the right medial gastrocnemius muscle, with electrical stimulation pads (2 × 4 in, PRO Advantage) secured both above and below the NIRS device. To reduce the impact of ambient light on the NIRS signal, the NIRS device was loosely wrapped with Coban. A thigh blood pressure cuff (Delfi Tourniquet Cuffs, Delfi Medical Innovations Inc, Vancouver, BC) was wrapped superior to the kneecap. After ensuring hemodynamic stability and collecting baseline data, 30 s of electrical stimulation was administered to increase the metabolic rate of the skeletal muscle. A 1‐min rest period was performed between the resting metabolism and the blood volume calibration. Electrical stimulation is turned on for 30 s and the cuff is rapidly inflated afterwards for at least 30 s but no longer than 1 min. A stable and flat total blood volume (tHb) signal should be achieved during this time and is later used in the analysis to correct for the initial change in blood volume when the cuff inflates. After correcting for blood volume, the physiological calibration is assessed by selecting the minimum and maximum during the ischemic calibration. Subsequently, a series of repeated arterial occlusions and reperfusions using the thigh blood pressure cuff was performed to assess the changes in oxygenated and deoxygenated haaemoglobin (HHb) via NIRS.

Utilising NIRS as a non‐invasive and reliable method to assess skeletal muscle mitochondrial capacity, the rate constant measures mitochondrial respiration and the ability of the muscle to replenish depleted ATP stores [38]. The NIRS device works by emitting light at two different wavelengths (760 and 850 nm) and three distances (30, 35 and 40 mm). The device measures oxygenated haemoglobin (O_2_Hb), HHb, difference in haemoglobin (Hb_DIFF_ = O_2_Hb−HHb), and total blood volume (tHb = O_2_Hb + HHb). Data were collected at 10 Hz and data analysis was performed using the Hb_DIFF_ signal. The slopes of each post‐electrical stimulation cuff occlusion were fit to a monoexponential curve to obtain a time constant (TC). The TC was then used to calculate the rate constant or oxidative capacity ([1/TC] × 60) as an index of skeletal muscle function. Two complete tests were conducted with at least 3 min of rest in between. The average of the two muscle function tests were used for analysis. Subsequently, calf adipose tissue thickness (ATT) was measured directly beneath the NIRS device (B‐mode ultrasound, LogiQ, GE Healthcare, Chicago, IL). Due to insufficient penetration of the NIR light into the gastrocnemius muscle, participants with an ATT greater than 30 mm were excluded from the study.

#### 2.3.3 Handgrip Strength

A small, handheld dynamometer (Lafayette Professional Grip Dynamometer, Lafayette, IN) was used to measure grip strength. Participants were asked to squeeze the instrument as hard as they can for 3–5 s, which measured the force applied in kilograms. Measurements were taken three times on the participant's dominant hand, and the highest of the values was used for analysis.

### 2.4 Assessment of Daily Physical Activity

All participants were asked to wear triaxial accelerometers (ActiGraph Model GT3X+, ActiGraph, Pensacola, FL) on their non‐dominant wrist continuously for 7 days while continuing with their normal activities. At the end of the 7‐day period, participants were instructed to return the accelerometer to the Georgia Prevention Institute using a postage paid envelope. All indices of physical activity were derived by processing raw accelerometer data (.gt3x) that were collected at 30 Hz time resolution. Briefly, the ActiLife software was used to convert the raw data to .csv files, which were processed with the open‐source R package GGIR (version 2.10.3) [40] for calibration to local gravity and calculation of non‐wear time. Non‐wear time was defined as periods of at least 60 consecutive minutes of low acceleration with little variability. The vector magnitude of the three axes was used to calculate activity‐related acceleration using Euclidian Norm minus 1 g [ENMO = √(*x* ^2^ + *y* ^2^ + *z* ^2^)−1]. For segments with missing data, the average of similar time‐of‐day data points from other days of measurement in the same individual were imputed. Data were initially aggregated in 5‐s time series. Participants were included if wear time was at least 16 h/day on at least 3 out of the 7 days with at least one complete 24‐h cycle recorded.

Physical activity parameters quantify the time spent in activities at different intensities during the day time (i.e., non‐sleeping period) using intensity thresholds [41]. The inactive time is defined as the total minutes with ENMO below 30 milligravity (mg), the light intensity physical activity is defined as the total minutes with ENMO between 30 and 100 mg, the moderate intensity physical activity is defined as the total minutes with ENMO between 100 mg and 400 mg, and the vigorous intensity physical activity is defined as the total minutes with ENMO higher than 400 mg.

### 2.5 Statistical Analysis

All analyses were performed using SPSS Statistics Version 29. Independent samples *t*‐tests were performed to identify group differences (i.e., overweight vs. obese) in demographics and clinical laboratory biomarkers. Univariate analysis of variance (ANOVA) was used to determine differences in SMOC and physical activity parameters between individuals who are overweight and obese with race and sex as controlled variables. Pearson's correlations were used to assess relationships between BMI, SMOC, and physical activity parameters. Effect sizes are reported as Cohen's *d* values to represent small (Cohen's *d* = 0.2), medium (Cohen's *d* = 0.5) and large (Cohen's *d* = 0.8) effect sizes [42]. Data are reported as mean ± standard error of mean (SEM) unless otherwise noted. Statistical significance (*) was set at *p* < 0.05.

## 3 Results

### 3.1 Participant Characteristics

Participant demographics and clinical lab values are presented in Table 1. As expected, individuals who were obese were heavier (*p* < 0.001) and exhibited a higher BMI (*p* < 0.001), percent body fat (*p* < 0.001) and visceral adipose tissue (*p* < 0.001) compared to individuals who were overweight. Fasting triglycerides (*p* = 0.021), fasting insulin (*p* < 0.001) and HOMA‐IR (*p* < 0.001) were significantly higher, and HDL cholesterol was lower (*p* < 0.001) in obese compared to individuals who were overweight. No other differences in participant characteristics or clinical laboratory values were found between groups (all *p* > 0.05).

**TABLE 1:** Participant characteristics and clinical laboratory values.

Variable	Overweight	Obese	*p* value
	(*n* = 73)	(*n* = 67)	
Demographic			
Sex (M/F)	24/49	30/37	0.148
Race (NHW/NHB)	39/34	30/37	0.308
Age (years)	34 ± 6	35 ± 6	0.350
Height (cm)	169 ± 10	171 ± 9	0.319
Weight (kg)	79 ± 9	103 ± 17	**<0.001**
BMI (kg/m^2^)	27.4 ± 1.5	35.2 ± 4.7	**<0.001**
Percent body fat (%)	35.9 ± 7.6	40.6 ± 7.1	**<0.001**
Fat free mass (kg)	50.9 ± 10.9	60.6 ± 11.3	**<0.001**
VAT (g)	495 ± 143	794 ± 259	**<0.001**
Clinical laboratory values			
TC (mg/dL)	182 ± 31	182 ± 34	0.870
HDL (mg/dL)	56 ± 15	48 ± 13	**<0.001**
LDL (mg/dL)	109 ± 30	114 ± 29	0.313
TRIG (mg/dL)	90 ± 63	115 ± 66	**0.020**
HbA_1c_ (%)	5.3 ± 0.3	5.3 ± 0.3	0.327
FBG (mg/L)	86 ± 9	89 ± 8	0.122
Insulin (uIU/mL)	9.2 ± 4.6	14.4 ± 8.3	**<0.001**
HOMA‐IR	1.99 ± 1.13	3.18 ± 1.96	**<0.001**

*Note:* Data are presented as mean ± SD; generalised estimating equations. Bold values indicate statistically significant differences between groups.

Abbreviations: BMI = body mass index, FBG = fasting blood glucose, HbA_1c_ = haaemoglobin A1c, HDL = high‐density lipoprotein, HOMA‐IR = homeostatic model assessment for insulin resistance, LDL = low‐density lipoprotein, TC = total cholesterol, TRIG = triglycerides, VAT = visceral adipose tissue.

### 3.2 Skeletal Muscle Function

**FIGURE 1:**
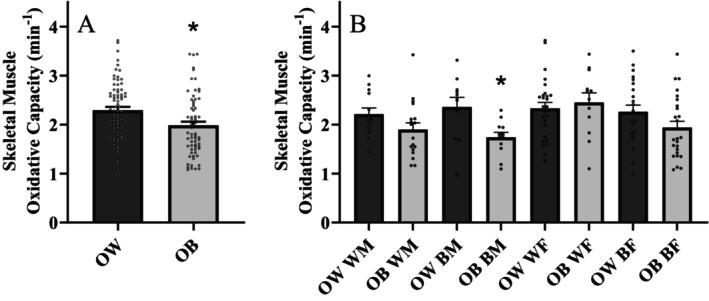
(A) Skeletal muscle oxidative capacity between overweight (OW) and obese (OB) individuals; *n* = 140 (overweight = 73, obese = 67) and (B) skeletal muscle oxidative capacity between overweight and obese individuals separated by race and sex; *n* = 140 (NHW males (WM) = 31, NHB males (BM) = 23, NHW females (WF) = 38, NHB females (BF) = 48). Independent samples *t* test. Data are presented as mean ± SEM. *indicates a significant difference from overweight.

Skeletal muscle endurance index was similar (*p* = 0.239; Cohen's *d* = 0.222) between individuals who were overweight (51.6 ± 20.0%) and obese (46.4 ± 20.6%). Figure 1A illustrates SMOC between groups. Individuals who were overweight exhibited a significantly (*p* = 0.004; Cohen's *d* = 0.501) higher SMOC compared to individuals who were obese. Overall, no differences between SMOC were observed between men and women (*p* = 0.095) or NHW and NHB (*p* = 0.183). There was no difference (*p* = 0.477; Cohen's *d* = −0.124) in handgrip strength between individuals who were overweight (38.6 ± 12.6 kg) and obese (40.1 ± 10.9 kg). Figure 1B presents SMOC broken down by sex and race in individuals who were overweight and obese. NHB men who were overweight exhibited a significantly (*p* = 0.010; Cohen's *d* = 1.185) greater SMOC compared to NHB men who were obese. No other sex and race differences in muscle oxidative capacity were observed between individuals who were overweight and obese. Handgrip strength was higher (*p* < 0.001; Cohen's *d* = 1.899) in men compared to women. There was no difference (*p* = 0.262) in handgrip strength between NHW and NHB individuals.

### 3.3 Daily Physical Activity

**FIGURE 2:**
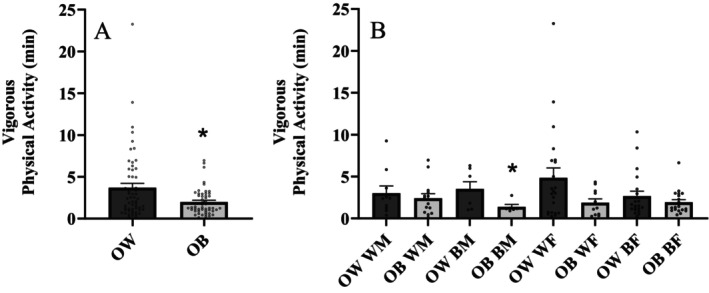
(A) Vigorous physical activity between overweight (OW) and obese (OB) individuals; *n* = 110 (overweight = 73, obese = 67) and (B) vigorous physical activity between overweight and obese individuals separated by race and sex; *n* = 140 (NHW males (WM) = 24, NHB males (BM) = 13, NHW females (WF) = 33, NHB females (BF) = 40). Independent samples *t* test. Data are presented as mean ± SEM. *indicates a significant difference from overweight.

Indices of physical activity in overweight and obese groups are presented in Table 2. Inactive time (*p* = 0.734), light physical activity (*p* = 0.217), moderate physical activity (*p* = 0.794), moderate‐vigorous physical activity (*p* = 0.979) and total activity time (*p* = 0.433) were all similar between groups. Overall, no differences between vigorous physical activity were observed between men and women (*p* = 0.560) or NHW and NHB (*p* = 0.103). Figure 2A illustrates vigorous physical activity between groups. Vigorous physical activity was significantly (*p* = 0.007; Cohen's *d* = 0.528) higher in individuals who were overweight compared to those who were obese. Figure 2B presents vigorous physical activity broken down by sex and race in individuals who were overweight and obese. Minutes spent conducting vigorous physical activity was significantly (*p* = 0.049; Cohen's *d* = 1.228) greater in NHB men who were overweight compared to NHB men who were obese. No other sex and race differences in physical activity were observed between individuals who were overweight and obese.

**TABLE 2:** Parameters of physical activity.

Variable	Overweight	Obese	*p* value
Inactive time (min)	662 ± 94	656 ± 91	0.733
Light physical activity (min)	234 ± 69	249 ± 58	0.214
Moderate physical activity (min)	82 ± 36	84 ± 38	0.793
MVPA (min)	86 ± 38	86 ± 38	0.979
Total physical activity (min)	320 ± 96	335 ± 84	0.374

*Note:* Values are mean ± SD; generalised estimating equations.

Abbreviation: MVPA = moderate‐to‐vigorous physical activity.

### 3.4 Relationships Among Adiposity, Physical Activity and Skeletal Muscle Oxidative Capacity

No significant correlations were observed between vigorous physical activity and SMOC (*r* = 0.027; *p* = 0.776), endurance index (*r* = 0.075; *p* = 0.495) or handgrip strength (*r* = 0.012; *p* = 0.904). While a significant negative relationship existed between BMI and vigorous physical activity (*r* = −0.218; *p* = 0.022), no such associations were found between BMI and SMOC (*r* = −0.157; *p* = 0.064) or endurance index (*r* = −0.081; *p* = 0.389). Although a nearly significant negative association was found between percent body fat and vigorous physical activity (*r* = −0.193; *p* = 0.051), no connections were observed with SMOC (*r* = 0.019; *p* = 0.826) or endurance index (*r* = 0.036; *p* = 0.714). There was a significant negative relationship between visceral adipose tissue and vigorous physical activity (*r* = −0.288; *p* = 0.003), but no correlations were found with SMOC (*r* = −0.122; *p* = 0.163) or endurance index (*r* = −0.099; *p* = 0.310). Within just the overweight group, there was a significant negative relationship between visceral adipose tissue and vigorous physical activity (*r* = −0.345; *p* = 0.010). However, no significant relationships between BMI, percent body fat, visceral adipose tissue, vigorous physical activity, SMOC or endurance index were observed within the obese group.

## 4 Discussion

The rates of overweight and obesity throughout the world continue to rise. It is well‐established that engaging in daily physical activity yields numerous health benefits, including mitigating the poor health outcomes associated with obesity. Indeed, physical activity plays a vital role in enhancing mitochondrial remodelling; however, prolonged physical inactivity can foster suboptimal mitochondrial function and instigate disease pathology. The intricate relationship between SMOC and physical activity is notably complex, especially when considering differences in adiposity, sex and race. There is compelling evidence that both physical activity and muscle oxidative capacity are greater in normal weight compared to overweight/obesity; however, there is scant data specifically comparing overweight to obesity. The prevalence of both NHB and individuals with greater adiposity within the Southeast region of the United States (where this study was conducted) presents a unique opportunity to investigate differences between overweight and obese individuals. Accordingly, findings from the present investigation demonstrate that compared to individuals who were obese, individuals who were overweight exhibit higher SMOC and participate in more vigorous physical activity. In addition, NHB men who are obese exhibit a lower SMOC compared to NHB men who are overweight. Although differences in muscle oxidative capacity and physical activity were observed between groups, there is no relationship between physical activity and oxidative capacity among individuals who were overweight and obese.

### 4.1 Adiposity and Skeletal Muscle Health

Skeletal muscle plays an important role in glucose homeostasis [43], metabolism [44], and cardiovascular disease prevention [45]. Indeed, increased adiposity can detrimentally affect skeletal muscle, manifesting as reduced muscle performance and lower skeletal muscle energetic efficiency and maximum oxidative capacity [46]. In the present study, individuals who were overweight exhibited greater SMOC compared to individuals who were obese. It is important to note that the differences in muscle oxidative capacity among individuals who were overweight and obese persisted even after controlling for both race and sex (*p* = 0.009). Endurance index, a test of muscle fatigability, and handgrip force, a test of muscle strength were both similar between overweight and obese groups. It is possible that these methods may not be sensitive enough to detect differences between groups; however, previous reports also demonstrate that both muscle fatigability [47, 48] and handgrip strength were similar between overweight and obesity [49]. In fact, no correlation between handgrip strength and SMOC was documented [50], which is consistent with the present findings. Despite this, both SMOC [51] and handgrip strength [52] are important measures of overall skeletal muscle health and should continue to be utilised, as they each represent independent risk factors of CVD. Nonetheless, the mechanisms contributing to differences in SMOC may be different from muscle endurance or muscle strength.

Although it may seem arbitrary, the difference in BMI between overweight and obesity likely has various health implications that may impact muscle oxidative capacity, but not other measurements of muscle health. Indeed, it is important to note that SMOC was non‐invasively assessed using NIRS, however, this novel technique is methodologically similar to the gold standard ^31^P‐MRS [39, 53, 54, 55] and has been used as a surrogate for skeletal muscle mitochondrial oxidative capacity. Certainly, ATT may influence SMOC. While initially the cut‐off for ATT was <3 cm, it is noteworthy that only eight participants exceeded an ATT of >1.5 cm. Even after excluding these individuals from the analysis, the overall findings remained consistent. Importantly, consideration should be taken when evaluating SMOC using NIRS in individuals who are morbidly obese as the increased ATT could limit skeletal muscle penetration and negatively influence the results. Furthermore, no discernible relationship between ATT and SMOC were observed in the present study. Although the present investigation cannot infer a causal relationship between adiposity and skeletal muscle health, it does provide the first evidence to suggest that muscle oxidative capacity is greater in overweight compared to obesity, albeit similar muscle endurance and muscle strength between groups. Accordingly, future studies are certainly needed to determine the intricate balance between muscle health and increased adiposity and determine the role that SMOC plays in the treatment and prevention of obesity. In addition, the current study uses BMI as a surrogate for adiposity. Consideration of central adiposity and fat‐free mass are needed in future studies as these indices of adiposity could contribute to the differences in SMOC between individuals who are overweight and obese. Further, future studies incorporating more conventional fitness measures (i.e., exercise capacity) to accompany skeletal muscle outcomes are certainly warranted.

Racial disparities in SMOC are evident with NHB individuals exhibiting greater SMOC compared to NHW individuals [22]. Although this previous data was conducted in a large adult population with a BMI between 18.5–39.9 kg/m^2^, differences in muscle oxidative capacity with respect to adiposity was not evaluated. Data from the present study demonstrate, overall, that there are no differences in SMOC between NHW and NHB individuals. However, within NHB men, only those men who were obese exhibited a lower SMOC compared with their overweight male counterparts. Indeed, there also appears to be a disparity in muscle oxidative capacity in NHB women; however this effect was not statistically significant (*p* = 0.093; Cohen's *d* = 0.495). Nonetheless, the overall differences in muscle oxidative capacity between overweight and obesity remained (*p* = 0.039) even with removal of NHB men from the analysis, indicating that disparities between individuals who were overweight and obese are not exclusively influenced by the racial disparity. While earlier research indicated that overall muscle size is greater in individuals who were NHB, the current data introduces a unique perspective of skeletal muscle health by highlighting a potential disparity specifically in skeletal muscle mitochondrial oxidative capacity.

### 4.2 Adiposity and Physical Activity

The well‐established benefits of daily physical activity include robust cardiovascular protection [56], metabolic and weight management [57] and overall increased longevity [58]. In the present investigation, individuals who were overweight engaged in a greater amount of vigorous physical activity compared to individuals who were obese, albeit a similar total time of overall, light and moderate physical activity. Existing literature indicates that there may be a connection between reduced physical activity and diminished muscle mass and strength [59]. The present findings, however, suggest that lower vigorous physical activity does not explain the lower SMOC observed in individuals who were obese. Indeed, the focus of the current study is comparing overweight and obesity, specifically in those without overt metabolic disease. As expected, individuals in the obese group were heavier, and had higher body fat and visceral adipose tissue. Importantly, glycemic control using HbA_1c_ was within normal limits and similar between groups, albeit individuals who were obese exhibited a significantly higher fasting insulin concentrations compared to the individuals who were overweight. Indeed, a negative relationship between vigorous physical activity and insulin concentrations (*r* = −0.227; *p* = 0.020) and HOMA‐IR (*r* = −0.224; *p* = 0.022) was observed. Despite these significant relationships, differences in vigorous physical activity persisted after controlling for circulating insulin (*p* = 0.048; Cohen's *d* = 0.397) and HOMA‐IR (*p* = 0.044; Cohen's *d* = 0.403). Additionally, there were no differences in the total daily physical activity between adiposity groups. These data suggest that it is not necessarily the duration of overall activity, rather the physical activity intensity that likely explains the observed differences in SMOC. Certainly, these findings warrant further investigation to fully comprehend the complexities of the relationship between adiposity and physical activity and its implications towards improved metabolic health.

There is evidence that NHW individuals participate in more physical activity compared to NHB individuals [13]. Despite this data reported previously from a large general population study, data from the present study demonstrates that in an overweight and obese cohort, there are no differences in vigorous physical activity between NHW and NHB individuals. However, NHB men who were obese engaged in less vigorous physical activity compared to NHB men who were overweight. In addition, NHW women who are overweight appear to engage in more (*p* = 0.091) vigorous physical activity compared to NHW women who are obese. Albeit these sex and race differences in vigorous physical activity, the overall finding that individuals who were obese engage in lower vigorous physical activity compared to those who are overweight persisted after controlling for sex and race (*p* = 0.011).

### 4.3 Relationships Among Adiposity, Physical Activity and Muscle Oxidative Capacity

It is important to note that the present study cannot infer a causal relationship between whether higher muscle oxidative capacity is preventing obesity or whether obesity is causing skeletal muscle dysfunction. Despite this, to our knowledge, no prior study has specifically examined the correlation between daily physical activity and SMOC between overweight and obese individuals. Even considering the broad range of BMI within the obese category (30.0–50.5 kg/m^2^) in the current study, no discernible relationship exists between BMI (or other indices of adiposity) and SMOC (*r* = 0.035; *p* = 0.777). In addition, no relationship between muscle oxidative capacity or muscle endurance and circulating concentrations of insulin or HOMA‐IR were identified, and differences in muscle oxidative capacity between groups persisted after controlling for insulin and HOMA‐IR (both *p* < 0.001). These findings suggest that the higher insulin observed in the obese group is not likely contributing to the lower muscle oxidative capacity that was observed. This finding is in line with a recent study that observed those with different insulin concentrations exhibited similar skeletal muscle mitochondrial function [60]. In the present study, there was also no relationship found between SMOC and visceral adipose tissue (*r* = −0.122; *p* = 0.163) or percent body fat (*r* = 0.019; *p* = 0.826), both important factors that play a role in obesity‐related health risks. Additionally, it is important to note that there was no relationship between BMI and vigorous physical activity (*r* = −0.033; *p* = 0.818); however, a significant negative relationship was observed between visceral adipose tissue and vigorous physical activity (*r* = −0.288; *p* = 0.003) and approaching significance using percent body fat (*r* = −0.193; *p* = 0.051) as a surrogate for adiposity. It is noteworthy that these patterns of inactivity are not likely an explanation for the lower SMOC that was observed.

### 4.4 Conclusion

In conclusion, both SMOC and vigorous physical activity were higher in individuals who were overweight compared to obese. NHB men who are overweight exhibit greater muscle oxidative capacity and engage in more vigorous physical activity compared to NHB men who are obese and suggest that the impact of obesity and physical activity on muscle health may manifest differently across racial groups. Nonetheless, no relationships were observed between indices of physical activity and SMOC assessed by NIRS in this overweight and obese cohort. This observation suggests that the higher vigorous physical activity observed in individuals who were overweight may not be the primary cause of the higher SMOC. Collectively, these data emphasise the need for more comprehensive investigations into the complex interactions between adiposity, physical activity, and muscle health with special consideration to sex and race.

## Author Contributions

A.B.S. analysed data, interpreted results of experiments, prepared figures, drafted manuscript, edited and revised manuscript, and approved final version of manuscript. C.C.D. performed experiments, analysed data, interpreted results of experiments, edited and revised manuscript, and approved final version of manuscript. J.C.L. performed experiments, edited and revised manuscript, and approved final version of manuscript. K.N. performed experiments, edited and revised manuscript, and approved final version of manuscript. X.W. conceived and designed research, analysed data, interpreted results of experiments, edited and revised manuscript, and approved final version of manuscript. R.A.H. conceived and designed research, analysed data, interpreted results of experiments, drafted manuscript, edited and revised manuscript, and approved final version of manuscript.

## Ethics Statement

All study protocols were approved by the Institutional Review Board at Augusta University.

## Conflicts of Interest

The authors declare no conflicts of interest.

## Data Availability

The datasets used during the current study are available from the corresponding author on reasonable request.
